# Management of Severe Ulcerative Colitis with Ambulatory Intravenous Corticosteroids (MOSAIC): A Treatment Approach to Avoid Hospitalization in Immunocompromised Patients

**DOI:** 10.1093/crocol/otaf053

**Published:** 2025-08-14

**Authors:** Sabrina L Chen, Nicole Arima, Kendall Beck, Uma Mahadevan, Sara Lewin

**Affiliations:** Department of Medicine, University of California, San Francisco, San Francisco, CA, United States; Division of Gastroenterology, Department of Medicine, University of California, San Francisco, San Francisco, CA, United States; Division of Gastroenterology, Department of Medicine, University of California, San Francisco, San Francisco, CA, United States; Division of Gastroenterology, Department of Medicine, University of California, San Francisco, San Francisco, CA, United States; Division of Gastroenterology, Department of Medicine, University of California, San Francisco, San Francisco, CA, United States

**Keywords:** acute severe ulcerative colitis (ASUC), ambulatory, quality of life, corticosteroid, COVID-19

## Abstract

**Introduction:**

Acute severe ulcerative colitis (ASUC) typically requires hospitalization for intravenous (IV) corticosteroid treatment and monitoring. In response to the need to reduce inpatient stays, especially during the COVID-19 pandemic, outpatient treatment models have gained interest. This study evaluated the feasibility, safety, and patient satisfaction of outpatient IV corticosteroid treatment for ASUC.

**Methods:**

We conducted a prospective cohort feasibility pilot study at a single academic center between May 2021 and October 2022. Fifteen adults with ASUC were enrolled and self-selected either outpatient or inpatient IV corticosteroid treatment. All participants received daily laboratory monitoring and symptom assessments for 14 days, with follow-up for 1 year. Primary outcomes included 90-day colectomy and 30-day readmission rates. Secondary outcomes included clinical activity scores, symptom and care satisfaction, and feasibility metrics.

**Results:**

Ten patients received outpatient care, and 5 were hospitalized. No patients in either group required colectomy within 90 days. One outpatient and no inpatients required colectomy within 1 year. Thirty-day readmission occurred in 30% of outpatients and 40% of inpatients. Clinical activity scores and satisfaction with food and sleep were similar between groups at baseline and on day 14. Initial care satisfaction was lower in the outpatient group but equalized by day 14. Outpatient care required significant coordination but was successfully delivered without adverse safety outcomes.

**Discussion:**

Outpatient IV corticosteroid treatment for select patients with ASUC is feasible, safe, and associated with comparable clinical outcomes and patient satisfaction compared to inpatient care. This model may offer a cost-effective alternative to hospitalization.

## Graphical abstract

**Graphical Abstract:**
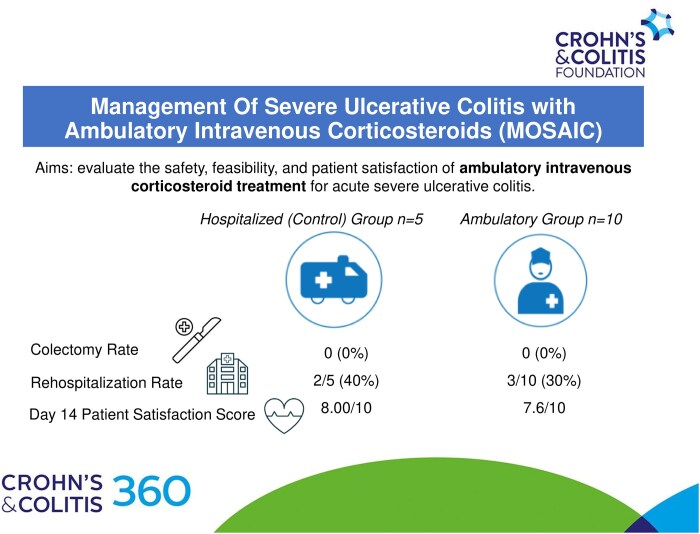

## Introduction

Acute severe ulcerative colitis (ASUC) affects up to 25% of individuals with ulcerative colitis and carries a mortality rate of approximately 1%.^1^^,^^2^ Historically, due to the high rate of morbidity and mortality in this population, hospital admission has been standard practice to exclude infective complications, receive gastroenterology, nutrition, and colorectal consultation, enabling endoscopic evaluation, and receiving intravenous (IV) corticosteroid and rescue therapy.^3^^,^^4^ ASUC-related healthcare costs remain high, primarily due to lengthy hospitalizations.^5^ Furthermore, high rates of depression, anxiety, and post-traumatic stress have been reported among patients with ASUC following hospital admissions.^6^ The COVID-19 pandemic has driven efforts to reduce inpatient stays, making ambulatory care pathways—with outpatient IV corticosteroids and medical assessment—a promising alternative.^5^

Sebastian et al.^7^ conducted the only existing study on ASUC management in ambulatory settings. Their multicenter post-hoc cohort study found comparable colectomy rates among UC patients managed entirely inpatient and those treated in the ambulatory setting (either entirely ambulatory or transitioned from the inpatient setting).

Adding to the existing evidence examining alternatives to hospitalization in ASUC, we report data on the feasibility pilot prospective cohort study, **M**anagement **O**f UC with **A**mbulatory **I**ntravenous **C**orticosteroids **(MOSAIC),** conducted at the University of California, San Francisco (UCSF). To the best of our knowledge, this is the first prospective study evaluating intensive outpatient management for ASUC requiring IV corticosteroids, as well as the first study to involve patient questionnaire data on overall satisfaction and quality-of-life metrics in this treatment setting.

## Methods

### Study cohorts

Our team conducted a prospective cohort feasibility pilot study, enrolling 15 ASUC patients from May 2021 to October 2022. Patients were recruited by direct provider referral, typically during a clinic visit or via electronic patient messaging, and self-selected inpatient or outpatient treatment plan after discussion with their provider.

Prior to initiation of the study, investigators worked with the UCSF infusion center to ensure a mechanism for timely scheduling of infusion appointments. Because IV corticosteroids did not require prior authorization from commercial insurers, insurance authorization was not a barrier. If a patient was unable to receive IV corticosteroids within 48 h, he or she was referred to the hospital for direct admission.

Inclusion criteria included patients aged 18 years and older with a diagnosis of moderate or severe ulcerative colitis (as defined by Truelove and Witts’ Criteria), who were nonresponsive to three days of oral prednisone 40 mg or greater (as determined by the clinical provider), and had pain manageable at home. Exclusion criteria included: active emesis, unable to maintain adequate oral intake (at least 1 L of fluid a day), and unable to receive care at a UCSF infusion center within 48 h.

Both hospitalized and ambulatory groups received baseline blood and stool testing, COVID-19 testing, and abdominal X-ray just prior to receiving IV steroids. The ambulatory group had an initial video visit with a gastroenterologist. All patients also received up to 7 days of IV steroids and daily labs (either in the hospital or at the UCSF infusion center), as well as a flexible sigmoidoscopy within 7 days. In the inpatient setting, patients received daily gastroenterology and hospitalist physician consultation, as well as nursing assessment multiple times per day. In the outpatient setting, patients could contact their outpatient gastroenterologist by telephone or electronic message as frequently as they liked.

### Data collection

An initial questionnaire was used to collect comprehensive patient data, including demographics, UC disease history (including previous medications, hospitalizations), as well as detailed medical and surgical histories. Subsequently, a Charlson Comorbidity Index was calculated for each patient. Most patients completed a daily survey for the first 14 days of the study. This daily survey included questions about food intake, sleep, abdominal pain, and red-flag symptoms that would necessitate hospital admission, satisfaction with the care plan, and components of the Simple Clinical Colitis Activity Index (SCCAI).^8^ Daily vitals, standard laboratory blood draws, and patient-reported symptoms were carefully documented and reviewed by the study team, notifying the treatment team of any significant abnormalities to support timely management. In the ambulatory setting, the treatment team worked closely with colorectal surgery colleagues to provide as-needed video visit consultations on an emergent basis.

After the initial 14 days of the study, patients were followed for at least 1 year. All questionnaires and clinical data were collected and entered into a secure, central REDCap (Research Electronic Data Capture) server.

### Outcomes of interest

The primary outcomes of interest were 90-day colectomy and 30-day readmission rates. Secondary outcome measures included duration of IV steroids; baseline and day 14 SCCAI scores; and day 14 food satisfaction, sleep satisfaction, and overall satisfaction scores (rated 0-10, with 0 being the worst satisfaction). This study was approved by the Institutional Review Board of UCSF (20-32474).

## Results

Of the 15 patients recruited, 10 patients opted to be treated with outpatient IV steroids with daily labs, while 5 patients were hospitalized and used as controls. All 15 patients were followed up weekly through day 14 of the study, with daily labs, vitals, and symptom check, and at 1 year.

Hospitalized and ambulatory groups were well matched for comorbidities, body mass index, years since UC diagnosis, history of prior UC-related hospitalization and UC severity (Table 1). Four of the 5 individuals in the hospitalized group (80%) and 8 of the 10 individuals in the ambulatory group (80%) met Truelove and Witt’s criteria for ASUC (Table 2). Ambulatory patients were younger, with a mean age of 38 vs 44 among those managed as inpatients (Table 1). The ambulatory group also had a higher proportion of male patients than the inpatient group (60% vs 20%, respectively). Additionally, the inpatient group had a lower proportion of patients with prior hospitalizations, 20% (*n* = 1) compared to 40% (*n* = 4) in the ambulatory group (Table 1).

**Table 1.** Baseline characteristics reported as mean (SD) for continuous variables and *n* (%) for discrete variables.

	Ambulatory IV steroids (*n* = 10)	Standard inpatient care (*n* = 5)
**Demographics**		
Age	38.34 (15.74)	43.80 (19.80)
Gender, male (%)	6 (60)	1 (20)
BMI	23.62 (3.13)	23.07 (2.94)
Charlson Comorbidity Index	0.50 (1.08)	0.20 (0.45)
Years since UC diagnosis	13.8 (12.48)	10.6 (11.84)
Number of patients with a prior hospitalization for ASUC	3 (30%)	1 (20%)
Endoscopic Mayo 3 disease	7 (70%)	4 (80%)
**Primary outcomes**		
90-day colectomy	0 (0%)	0 (0%)
30-day readmission	3/10 (30%)	2/5 (40%)
Secondary outcomes		
Duration of IV steroids (days)	5.3 (1.33)	5.0 (1.87)
Baseline SCCAI score	11.90 (3.34)	12.80 (1.10)
Day 14 SCCAI score	7.14 (2.34)	6.75 (2.50)
Baseline food satisfaction score (0-10)	3.88 (1.13)	3.60 (0.89)
Day 14 food satisfaction score (0-10)	4.14 (0.89)	4 (1)
Baseline sleep satisfaction score (0-10)	2.38 (0.92)	3 (0.71)
Day 14 sleep satisfaction score (0-10)	2.71 (0.49)	2.43 (0.82)
Baseline care satisfaction score (0-10)	6.38 (2.45)	9.40 (0.55)
Day 14 care satisfaction score (0-10)	7.57 (2.23)	8.00 (2.45)

Abbreviations: ASUC, acute colitis; BMI, body mass index; IV, intravenous; SCCAI, Simple Clinical Colitis Activity Index; UC, ulcerative colitis.

**Table 2.** Individual characteristics for 5 hospitalized patients and 10 ambulatory patients at baseline (day 1 of study).

	Age	Disease duration (years)	Gen-der	BM (per day)	Visible Blood in Stool	Temp (°F)	HR (BPM)	ESR (mm/h)	FCP (μg/g)	Hgb (g/dL)	Truelove and Witts’ criteria
**Control 1**	47	3	F	8	Yes	36.4	106	105	1440	9.2	Severe
**Control 2**	28	2	F	7	Yes	36.8	122	19	NA	9.2	Severe
**Control 3**	72	22	F	8	Yes	37.0	77	52	104	10.6	Severe
**Control 4**	50	25	M	6	Yes	36.8	107	12	524	15.4	Severe
**Control 5**	22	1	F	8	Yes	36.7	93	2	182	13.5	Moderate
**Ambulatory 1**	61	15	M	8	Yes	37.8	55	41	596	11.4	Severe
**Ambulatory 2**	22	2	M	8	Yes	37.2	73	20	552	12.7	Moderate
**Ambulatory 3**	34	19	M	12	Yes	36.8	72	31	1730	12.6	Severe
**Ambulatory 4**	35	8	M	20	Yes	36.9	83	62	3029	13.2	Severe
**Ambulatory 5**	38	13	F	15	Yes	36.6	90	35	400	11.7	Severe
**Ambulatory 6**	71	44	F	6	Yes	36.2	84	92	1560	10.8	Severe
**Ambulatory 7**	25	20	M	10	Yes	36.7	116	25	>3000	8.7	Severe
**Ambulatory 8**	27	11	F	7	Yes	37.1	65	25	928	10.5	Severe
**Ambulatory 9**	33	5	M	5	Yes	36.3	66	15	>3000	13.4	Moderate
**Ambulatory 10**	34	1	F	9	Yes	36.9	83	5	2280	10.2	Severe

	Endoscopic severity*	Day 1 SCCAI	Day 14 SCCAI	Day 14 Food Satisfaction	Day 14 Sleep Satisfaction	Day 14 Overall Care Satisfaction	90-day Readmission
**Control 1**	MAYO 3	12	6	4	3	10	No
**Control 2**	MAYO 3	12	7	5	2	10	Yes
**Control 3**	MAYO 2	14	N/A	N/A	N/A	N/A	Yes
**Control 4**	MAYO 3	12	4	4	1	5	No
**Control 5**	MAYO 3	14	10	3	2	7	No
**Ambulatory 1**	MAYO 3	6	8	4	3	6	Yes
**Ambulatory 2**	MAYO 3	10	5	4	3	6	No
**Ambulatory 3**	MAYO 2	14	N/A	N/A	N/A	N/A	No
**Ambulatory 4**	MAYO 3	15	7	5	2	10	No
**Ambulatory 5**	MAYO 2	15	N/A	N/A	N/A	N/A	No
**Ambulatory 6**	MAYO 2	11	9	5	3	9	No
**Ambulatory 7**	MAYO 3	12	N/A	N/A	N/A	N/A	No
**Ambulatory 8**	MAYO 2	15	3	5	3	9	No
**Ambulatory 9**	MAYO 3	7	9	3	2	9	Yes
**Ambulatory 10**	MAYO 3	14	9	3	3	4	Yes

a Endoscopic severity is reported from the most recent endoscopic evaluation to day 1 of IV corticosteroid start.

N/A represents patients who had missing data at the time point.

Abbreviations: Alb, albumin; BM, bowel movements; BPM, beats per minute; ESR, erythrocyte sedimentation rate; FCP, fecal calprotectin; Hgb, hemoglobin; HR, heart rate; Temp, temperature; SCCAI, Simple Clinical Colitis Activity Index.

No patients in either cohort required colectomy within 90 days of admission. In addition, those in the ambulatory cohort had a numerically lower rate of 30-day readmission (3 of 10, 30%) compared with the inpatient cohort (2 of 5, 40%). One patient in the ambulatory group (as opposed to none in the inpatient group) required colectomy within 1 year of study enrollment. One-year readmission rates remained similar between the 2 groups (30% for the ambulatory group, 40% for inpatient group) (Table 1).

Both groups had similar baseline (11.9 for the ambulatory group and 12.8 for the inpatient group) as well as day 14 SCCAI scores (7.14 for the ambulatory group and 6.75 for the inpatient group). In addition, both groups had similar baseline and day 14 food/sleep satisfaction scores. While initial care satisfaction was lower in the ambulatory group (6.38 rather than 9.4 in hospitalized group), the day 14 care satisfaction score was similar between the two groups (Table 1).

## Discussion

In conclusion, our findings support that in patients with ASUC, outpatient treatment with IV corticosteroids and outpatient coordination of care can be delivered safely and effectively, with similar rates of patient care satisfaction. Although the study was underpowered for our primary and secondary outcomes, we report no numerical difference in the requirement for 90-day colectomy among ASUC patients treated in the ambulatory setting and no difference in 30-day rehospitalization rates for further flares. Furthermore, both average baseline and day 14 SCCAI scores were similar between groups. Initially, patient care satisfaction was higher in the inpatient group, likely due to closer medication management and fewer external challenges, such as the need to travel to an infusion center or manage pain at home—factors that can be particularly challenging during the early, more severe phase of a flare. However, by day 14, care satisfaction levels were similar between both groups. Moving forward, improvements in outpatient care delivery may help to close this gap in patient experience within the ambulatory arm. While there has been anecdotal success in treating patients with ASUC in the ambulatory setting, this is, to the best of our knowledge, the first prospective cohort study comparing ambulatory ASUC treatment with traditional inpatient pathways.

This was a proof-of-concept feasibility study with several limitations. First, the sample size was too small to detect significant differences between groups. Second, recruitment was based on direct provider referrals rather than universal screening, which may limit generalizability and introduce self-selection bias, as patients chose between hospitalization and ambulatory care after discussion with their providers. Importantly, this study is also subject to confounding by indication, whereby patients perceived to be more ill—either by themselves or their treating physicians—were preferentially referred to inpatient care. This likely led to the hospitalization of patients with more severe symptoms or higher perceived risk, and the selection of comparatively healthier or more stable individuals for outpatient treatment, potentially biasing clinical outcomes in favor of the ambulatory group. Additional confounding may have occurred between enrolled and non-enrolled patients, as only those referred and consenting were included; a more comprehensive study design would involve intercepting all ASUC patients at the time of emergency department presentation or referral for admission and randomizing them to either ambulatory or inpatient care. In addition, follow-up survey data were limited. Despite automated emails and follow-up calls from our study team, patients did not necessarily respond to all survey data. Notably, while the average age of the ambulatory group was slightly younger (38 vs 44), baseline disease severity, endoscopic severity, and SCCAI scores were similar between both groups. Although our current data cannot be used to define a suitable cohort for ambulatory ASUC management, we hypothesize that younger, motivated, and engaged patients may be more appropriate for this pathway. Further study with a prospective randomized multi-center study design would best address these limitations.

Additionally, the care delivery for the ambulatory group required significant administrative coordination to ensure all components were in place—an infrastructure that is more seamlessly integrated within the hospital setting. To scale this model, a dedicated study team, administrative support, reserved clinical time, and rapid access to endoscopy would be essential. Just as critical to broader implementation is the establishment of an expedited pathway for maintenance therapy initiation. In our study, outpatient treatment teams were able to initiate rescue and maintenance therapies without requiring hospitalization, utilizing rapid prior authorization processes and close coordination with infusion and pharmacy services. Although we did not conduct a formal cost analysis, given the high cost of hospitalization, a scaled version of this program would likely be cost-effective. In addition to potential cost savings, ambulatory management of ASUC may help mitigate the psychological burden associated with hospitalization. While we did not assess mental health outcomes directly, our findings showed comparable patient satisfaction scores between ambulatory and hospitalized groups by day 14—driven by an improvement in satisfaction among ambulatory patients and a decline among those hospitalized. Prior studies have similarly reported that patients hospitalized for ASUC often express a strong desire to avoid inpatient care, citing prior negative experiences, emotional distress, and logistical challenges such as distance from home, work disruptions, and caregiving responsibilities.^9^ These observations suggest that ambulatory care models may not only be clinically effective but also better aligned with patient preferences and overall psychosocial well-being.

This pilot also revealed several operational and logistical challenges that will inform future implementation. Coordinating daily lab monitoring, infusion scheduling, and timely endoscopy access required substantial effort. Moreover, patient engagement and proactive communication proved to be vital to ensuring safety and adherence in the outpatient setting. These lessons emphasize the need for integrated infrastructure, streamlined workflows, and 24/7 study coordination to support a broader rollout or a future prospective randomized trial.

Despite these limitations, our study captures an emerging practice that challenges conventional treatment paradigms. We successfully provided expedited outpatient care through the infusion center and specialty consultation as needed without discernible differences in patient outcomes or patient satisfaction.

## Funding

The authors did not receive support from any organization for the submitted work.

## Conflicts of Interest

None declared.
